# Significance of measuring the severity of emphysema, in combination with spirometry, on the risk evaluation of patients undergoing major lung resection for cancer^†^

**DOI:** 10.1093/icvts/ivaf027

**Published:** 2025-02-11

**Authors:** Souichi Suzuki, Aya Harada-Takeda, Shoichiro Morizono, Koji Takumi, Tadashi Umehara, Go Kamimura, Masaya Aoki, Toshiyuki Nagata, Kazuhiro Ueda

**Affiliations:** Department of General Thoracic Surgery, Kagoshima University Graduate School of Medical and Dental Sciences, Kagoshima, Japan; Department of General Thoracic Surgery, Kagoshima University Graduate School of Medical and Dental Sciences, Kagoshima, Japan; Department of General Thoracic Surgery, Kagoshima University Graduate School of Medical and Dental Sciences, Kagoshima, Japan; Department of Radiology, Kagoshima University Graduate School of Medical and Dental Sciences, Kagoshima, Japan; Department of General Thoracic Surgery, Kagoshima University Graduate School of Medical and Dental Sciences, Kagoshima, Japan; Department of General Thoracic Surgery, Kagoshima University Graduate School of Medical and Dental Sciences, Kagoshima, Japan; Department of General Thoracic Surgery, Kagoshima University Graduate School of Medical and Dental Sciences, Kagoshima, Japan; Department of General Thoracic Surgery, Kagoshima University Graduate School of Medical and Dental Sciences, Kagoshima, Japan; Department of General Thoracic Surgery, Kagoshima University Graduate School of Medical and Dental Sciences, Kagoshima, Japan

**Keywords:** low-attenuation area, postoperative cardiovascular complication, lung cancer

## Abstract

**OBJECTIVES:**

Predicted postoperative forced expiratory volume in 1 s (ppoFEV1) and predicted postoperative diffusing capacity of the lungs for carbon monoxide (ppoDLco) are the two most significant parameters for predicting the risk of cardiopulmonary complications after major lung resection for lung cancer. Although the severity of pulmonary emphysema on computed tomography may be an important risk factor for postoperative complications, the clinical significance of measuring the severity of pulmonary emphysema, in combination with ppoFEV1 and ppoDLco, has never been evaluated.

**METHODS:**

We measured the severity of pulmonary emphysema, representing the percentage of low-attenuation area (< −950 Hounsfield units), in the whole lung field, in addition to ppoFEV1 and ppoDLco, in 451 patients who underwent major lung resection for primary lung cancer. We also measured the volume of the upper and lower lobes of emphysematous and non-emphysematous lungs.

**RESULTS:**

Postoperative cardiopulmonary complications developed in 60 patients (13.3%). According to a receiver operating characteristic curve analysis for the diagnostic potential of postoperative complications, the area under the curve was highest for the severity of emphysema, followed by ppoDLco and ppoFEV1. According to a stepwise multivariable logistic regression analysis, the severity of emphysema and ppoDLco was identified as independent risk factors for postoperative complications. Neither the heterogeneous distribution of emphysema nor that of non-emphysema was associated with the occurrence of complications in patients with upper lobe disease or in patients with lower lobe disease.

**CONCLUSIONS:**

The severity of emphysema on computed tomography is a relevant risk factor for cardiopulmonary complications after major lung resection.

**Clinical Registration Number:**

The study was approved by our institutional review board of No. 240077.

## GRAPHICAL ABSTRACT

**Figure:**
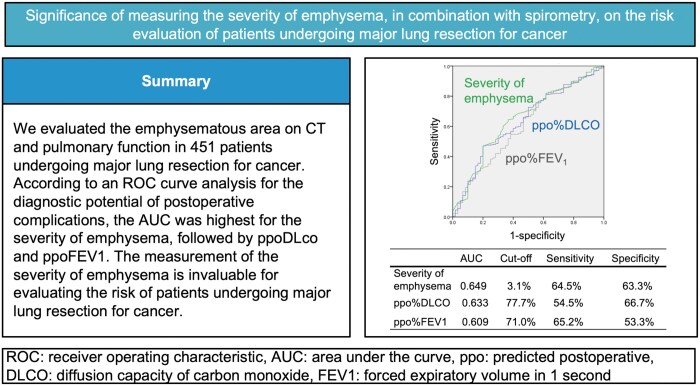

## INTRODUCTION

Major lung resection is the most curative option for clinically diagnosed stage I and II lung cancer. However, a proportion of patients experience postoperative cardiopulmonary complications (CPCs), which adversely affect their quality of life, prevent early postoperative rehabilitation and are associated with a higher incidence of postoperative cancer recurrence [1, 2]. Therefore, accurate identification of the risk of postoperative CPCs is indispensable for preoperative counselling. According to the relevant literature, the routine measurement of forced expiratory volume in 1 s (FEV1), forced vital capacity (FVC) and diffusing capacity of the lungs for carbon monoxide (DLCO) is recommended in preoperative patients undergoing major lung resection to screen for the risk of CPCs [2–9]. In particular, the percentage of predicted postoperative FEV1 (ppo%FEV1) and the percentage of predicted postoperative DLCO (ppo%DLCO), representing the postoperative pulmonary functional reserve, are the fulcral parameters for predicting the occurrence of postoperative CPCs [10–13]. However, spirometry assessment cannot be applied to all patients undergoing surgery for lung cancer because the results of spirometry depend on the patient’s efforts. In contrast, whole-lung computed tomography (CT) is routinely performed in patients with lung cancer for the staging of lung cancer. CT is also beneficial for diagnosing the severity and distribution of underlying lung diseases such as pulmonary emphysema. Some investigators have assessed the impact of the severity of emphysema on early outcomes after major lung resection for lung cancer. They reported that patients with severe emphysema are likely to develop postoperative air leaks [14], hypoxemia [15 ] and CPCs [16–22]. Unfortunately, none of the investigators assessed the significance of the severity of emphysema on the development of CPC, together with ppo%FEV1 and ppo%DLCO. In addition, although pulmonary emphysema is often heterogeneously distributed, the significance of lung heterogeneity to the development of CPCs remains unclear. The aim of the present study was to clarify the significance of the severity of emphysema, in addition to ppo%FEV1 and ppo%DLCO, in preoperative risk assessments for patients with primary lung cancer. In addition, we aimed to clarify the significance of lung heterogeneity in preoperative risk assessments for primary lung cancer.

## PATIENTS AND METHODS

### Patients

This study was approved by our Institutional Review Board (No. 240077E) on 12th September 2024, and informed consent of patients was waived. We retrospectively reviewed 451 consecutive patients who underwent major lung resection (anatomical segmentectomy or lobectomy) for clinically diagnosed primary lung cancer at Kagoshima University Hospital between January 2016 and December 2021. Surgery was done via open thoracotomy (*n* = 165), video-assisted thoracic surgery (VATS; *n* = 237) or robot-assisted thoracic surgery (RATS; *n* = 49). All patients who smoked stopped smoking for at least 3 weeks before surgery. The oncologic status was diagnosed based on the eighth edition of the TNM staging system. Tumour location was classified as either upper site (upper lobe or middle lobe) or lower site (lower lobe). Surgery was performed via open thoracotomy (*n* = 165), VATS (*n* = 237) or RATS (*n* = 49). The patient characteristics are shown in Table 1.

**Table 1:** Patient characteristics according to presence or absence of postoperative complications

Variables	Total (*n* = 451)	Complication		*P*
		No (*n* = 391)	Yes (*n* = 60)	
Sex (M/F)	245/206	201/190	44/16	0.002
Laterality (right/left)	269/182	233/158	36/24	1
Tumour site (upper/lower)	287/164	258/133	29/31	0.014
Procedure (Seg/Lob)	59/392	52/339	7/53	0.839
Approach (open/VATS or RATS)	165/286	138/253	27/33	0.100
Resected segments	3.8 ± 1.5	3.7 ± 1.5	4.0 ± 1.4	0.174
Pack-year smoked	25.4 ± 31.3	24.0 ± 31.0	34.4 ± 30.0	0.017
Consolidation tumour size (mm)	20.9 ± 15.2	20.6 ± 15.2	22.8 ± 15.7	0.297
%DLCO	100.2 ± 24.2	101.5 ± 24.5	92.2 ± 21.0	0.006
%FEV1	94.1 ± 20.2	95.0 ± 19.8	88.3 ± 22.2	0.018
FEV1/FVC (%)	73.6 ± 9.7	74.1 ± 9.3	70.6 ± 11.8	0.034
%FVC	102.3 ± 16.5	102.8 ± 16.3	99.7 ± 17.3	0.173
ppo%FEV1	75.4 ± 17.7	76.3 ± 17.5	69.4 ± 18.0	0.005
ppo%DLCO	80.4 ± 21.1	81.5 ± 21.1	73.0 ± 19.7	0.003
%LAA	5.77 ± 8.44	5.35 ± 8.07	9.19 ± 10.36	0.008
%LAA (≥19%/<19%)	90/361	69/322	21/39	0.003

The values are expressed as the mean ± standard deviation (SD).

Seg: segmentectomy; Lob: lobectomy.

All patients received a prophylactic infusion of antibiotics, mainly cefazolin sodium, immediately before the operation and every 3 hours during the operation. All patients underwent hilar and mediastinal lymph node dissection. We routinely dissected the superior mediastinal nodes for upper lobe cancer and inferior mediastinal nodes for lower lobe cancer, whereas we dissected both the superior and inferior mediastinal nodes for middle lobe cancer.

### Postoperative complications

Postoperative CPCs included pneumonia, broncho-pleural fistula, atelectasis, empyema, arrhythmia, respiratory failure, pulmonary vein thrombus, pleural effusion requiring tube drainage, acute exacerbation of interstitial pneumonia and prolonged air leak (≥ 5 days), which developed within 30 days after surgery. Postoperative CPCs were graded based on the Clavien–Dindo grading system and were recorded prospectively in our database [23, 24].

### Computed tomography

Helical CT scans were obtained using multidetector row CT scanners (IQon spectral CT, Philips Healthcare, Best, the Netherlands; or SOMATOM Force, Siemens Healthcare, Forchheim, Germany). All patients were scanned in the supine position with their hands raised above the heads. Imaging parameters were as follows: tube voltage, 120 kVp; gantry rotation time, 0.4 or 0.5 s; effective tube current-time product, 160 mAs with auto-modulation; pitch, 0.703 or 1.2; and detector-row configuration, 64 × 0.625 or 96 × 0.6 mm. Threshold limits of −600 to −1024 HU were applied to segment the entire lung and exclude soft tissues surrounding the lungs as well as large vessels, atelectasis, fibrosis and tumours within the lung. The volume of a lung segmented by certain threshold limits can be readily obtained using an imaging software program (Fuji, SYNAPSE VINCENT, Tokyo, Japan). We defined the percentage of low-attenuation area (%LAA) as the proportion of emphysematous lung volume (< −950 HU) to the total lung volume (−600 to −1024 HU), as reported previously [25].

### Assessment of lung anatomical heterogeneity

To assess the lung anatomical heterogeneity, we divided the bilateral lung into the upper portion (bilateral upper and middle lobes) and lower portion (bilateral lower lobe). Because emphysema is often distributed heterogeneously to the upper or lower lung portion, we measured %LAA in the upper and lower portions individually (%LAA_upper_ and %LAA_lower_). Likewise, because the relative volumes of the upper and lower portions differed by various degrees in each patient, we measured the volume of the upper and lower portions individually. To measure the functioning volume of the upper and lower portions, we excluded the volume of the emphysematous area (< −950 HU). Finally, we calculated the functioning lung volume of the upper portion (per segment) and the functioning lung volume of the lower portion (per segment).

### Statistical analysis

Values are expressed as the mean ± standard deviation (SD). To compare patients with and without CPC, the unpaired Student’s *t-*test was used to test continuous variables, while Fisher’s exact test was used to compare the categorical variables. A stepwise multivariable logistic regression analysis was used to determine independent predictors of CPC. Receiver operating characteristic (ROC) curves were constructed to determine the diagnostic performance of CPCs among various parameters by calculating the area under the ROC curve (AUC). The difference of AUCs between the groups was analyzed by DeLong’s test. *P* values of <0.05 were considered to indicate statistical significance. Statistical analyses were performed using the SPSS software program (SPSS Statistics version 22; IBM, USA).

## RESULTS

### Postoperative complications

Postoperative CPCs developed in 60 (13.3%) patients. The most frequent complication was prolonged air leak, followed by pneumonia, broncho-pleural fistula, atelectasis, empyema, arrhythmia, respiratory failure, pulmonary vein thrombus, pleural effusion requiring tube drainage and acute exacerbation of interstitial pneumonia (Table 2). There were two in-hospital deaths (0.4%); the cause of death was an acute exacerbation of interstitial pneumonia in one case and bronchopleural fistula in one case. Clavien–Dindo grade ≥III postoperative CPCs developed in 46 patients (10.2%).

**Table 2:** Postoperative cardiopulmonary complications (*n* = 60)

Variables		*n*
Complications		
	Prolonged air leak	24
	Pneumonia	16
	Bronchopleural fistula	6
	Atelectasis	5
	Empyema	3
	Arrythmia	2
	Respiratory failure	1
	Pulmonary vein thrombus	1
	Pleural effusion	1
	Interstitial pneumonia	1
Clavien–Dindo grade		
	II	14
	IIIa	35
	IIIb	9
	IV	0
	V	2
Total		60

### Predictors of postoperative CPCs

**Figure 1:**
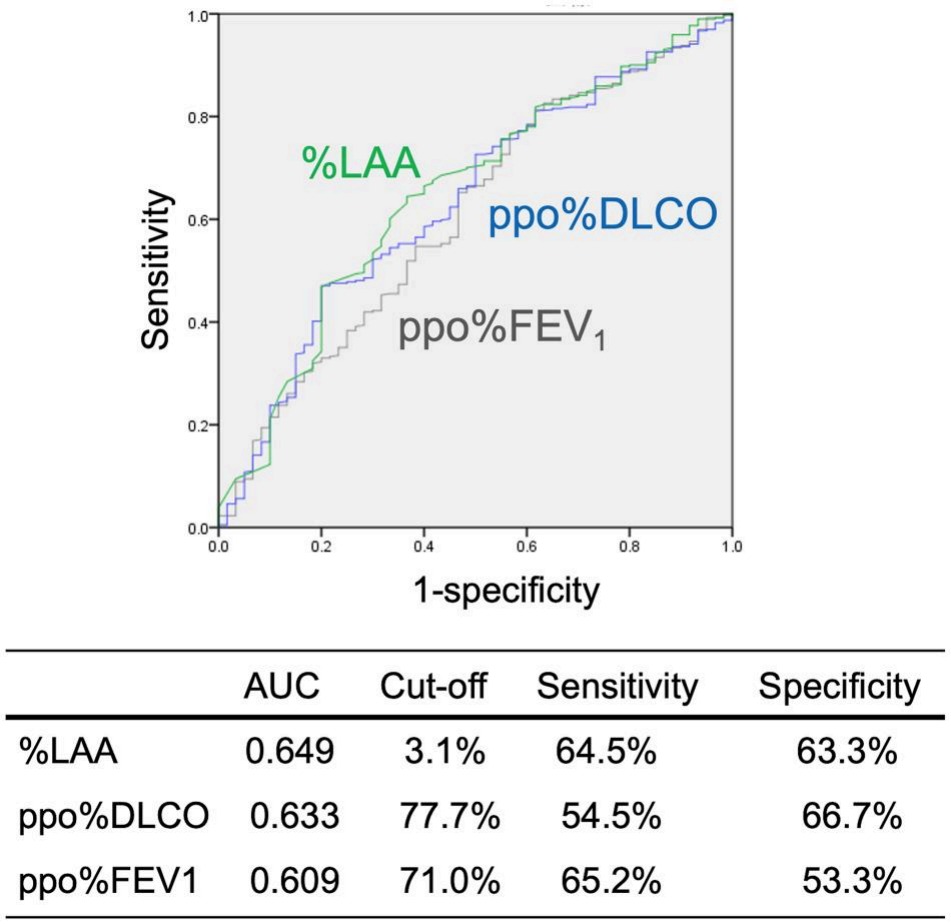
ROC curves showing potential of %LAA, ppo%DLCO and ppo%FEV1 for predicting postoperative complications. The area under the curve was highest for %LAA, followed by ppo%DLCO and ppo%FEV1, regardless of no statistical differences between the groups

**Figure 2:**
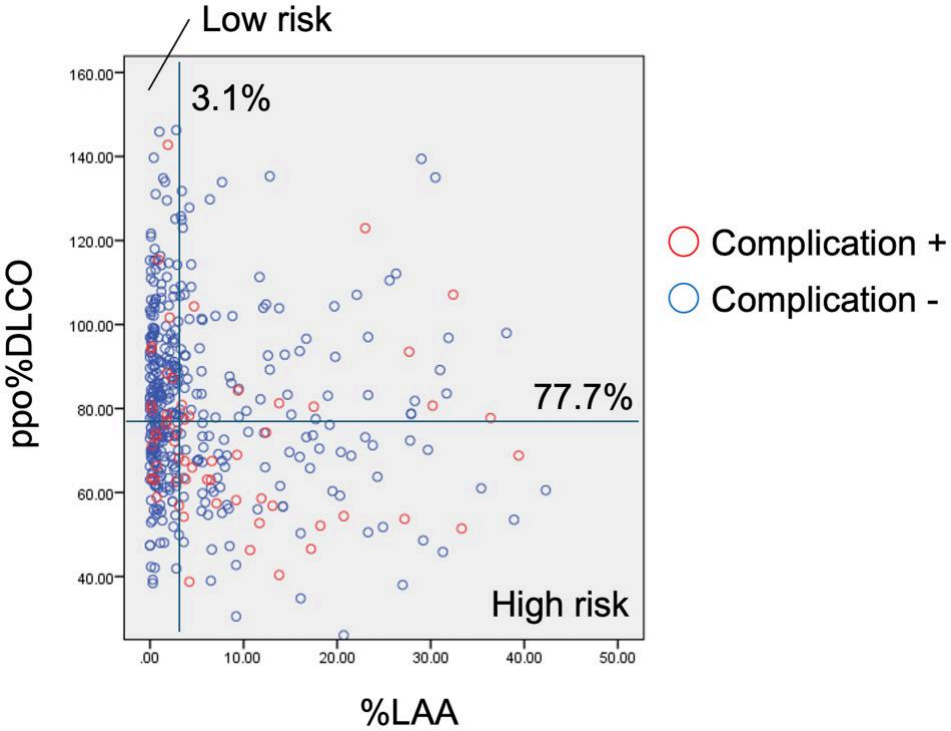
Scatter plot of %LAA and ppo%DLCO. Patients with postoperative complication are shown in red open circles. Note that patients with postoperative complication are concentrated in high-risk area (both ppo%DLCO and %LAA are above cut-off values)

Postoperative CPCs developed predominantly in male patients (*P* = 0.002), patients with more cigarettes smoked (*P* = 0.017) and patients with lower-lobe disease (*P* = 0.014). Patients who developed postoperative CPCs showed lower preoperative pulmonary function test results with respect to %FEV1, FEV1/FVC, ppo%FEV1, %DLCO and ppo%DLCO, in comparison to patients who did not (Table 1). In addition, patients who developed postoperative CPCs showed a higher %LAA than those who did not (Table 1). According to the stepwise multivariable logistic regression analysis with ppo%FEV1, ppo%DLCO and %LAA, ppo%DLCO (HR 0.981, 95% confidence interval [CI]: 0.967–0.995, *P* = 0.010) and %LAA (HR 1.039, 95% CI: 1.011–1.068, *P* = 0.006) were identified as independent predictors of postoperative CPCs (Table 3). According to the ROC curve analysis to identify the diagnostic potential of ppo%FEV1, ppo%DLCO and %LAA, the area under the curve was highest for %LAA (0.649), followed by ppo%DLCO (0.633), and ppo%FEV1 (0.609), regardless of no statistical differences between the groups (Fig. 1). The cutoff value, sensitivity and specificity for ppo%FEV1, ppo%DLCO and %LAA are also shown in Fig. 1. A scatter plot of %LAA vs ppo%DLCO is shown in Fig. 2, where postoperative CPCs was concentrated in ppo%DLCO < 77.7% and %LAA > 3.1%.

**Table 3:** Univariable and stepwise multivariable logistic regression analysis for postoperative cardiopulmonary complication (*n* = 451)

	Univariable analysis			Multivariable analysis		
Variables	RR	95% CI	*P*	RR	95% CI	*P*
ppo%DLCO	0.979	0.965–0.003	0.004	0.981	0.967–0.995	0.010
%LAA	1.042	1.014–1.070	0.003	1.039	1.011–1.068	0.006
ppo%FEV1	0.978	0.962–0.993	0.006	–	–	–

RR: relative risk.

### Factors associated with %LAA

According to the correlation analysis, %LAA was only weakly correlated with FEV1/FVC (*r* = −0.336), %FEV1 (*r* = −0.263) and pack-year smoking (*r *= 0.213), and %LAA was very weakly correlated with %DLCO (*r* = −0.124), body mass index (*r* = −0.187) and age (*r* = −0.014) (Supplementary Fig. S1).

### Assessment of lung anatomical heterogeneity

**Figure 3:**
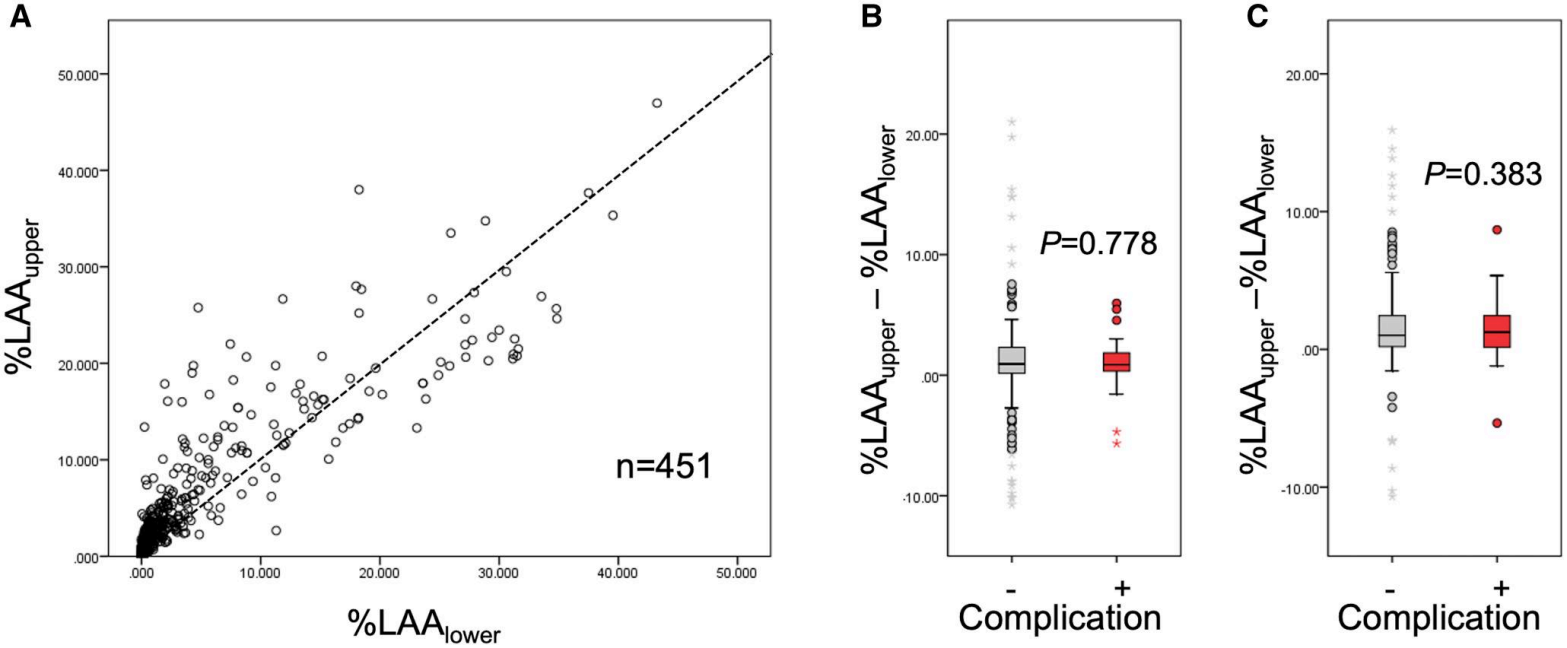
Scatter plot of %LAA for bilateral upper lobe (%LAA_upper_) and %LAA for bilateral lower lobe (%LAA_lower_) (**A**). (%LAA_upper_–%LAA_lower_)/segment is not significantly different between patients with postoperative complication and those without, when analysis is restricted in patients undergoing upper lobectomy (**B**). Likewise, (%LAA_upper_–%LAA_lower_)/segment is not significantly different between patients with postoperative complication and those without, when analysis is restricted in patients undergoing lower lobectomy (**C**)

**Figure 4:**
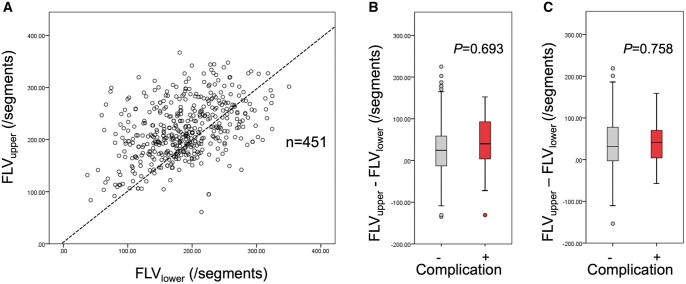
Scatter plot of functioning lung volume (FLV) per segment for bilateral upper lobe (FLV_upper_) and FLV per segment for bilateral lower lobe (FLV_lower_) (**A**). (FLV_upper—_FLV_lower_)/segment is not significantly different between patients with postoperative complication and those without when analysis is restricted in patients undergoing upper lobectomy (**B**). Likewise, (FLV_upper—_FLV_lower_)/segment is not significantly different between patients with postoperative complication and those without when analysis is restricted in patients undergoing lower lobectomy (**C**)

Figure 3A shows a scatter plot of %LAA_upper_ vs %LAA_lower_, showing patients with upper lobe-dominant emphysema, lower lobe-dominant emphysema and diffuse emphysema. Because upper lobectomy can strongly impact early postoperative outcomes in patients with lower lobe-dominant emphysema, we compared %LAA_upper_–%LAA_lower_ between patients with and without postoperative CPCs among patients undergoing upper lobectomy. As a result, %LAA_upper_–%LAA_lower_ was comparable between the two groups (Fig. 3B). Likewise, %LAA_upper_–%LAA_lower_ was comparable between the two groups in patients undergoing lower lobectomy (Fig. 3C). Figure 4A shows a scatter plot of the functioning volume of the upper portion (/segment) vs the functioning volume of the lower portion (/segment), showing patients with upper lobe-dominant lung, lower lobe-dominant lung and equally sized lung. Because upper lobectomy can strongly affect early postoperative outcomes in patients with upper lobe-dominant lung, we compared the functioning volume of the upper portion (/segment)—the functioning volume of the lower portion (/segment) between patients with and without postoperative CPCs among patients undergoing upper lobectomy. The functioning volume of the upper portion (/segment)—the functioning volume of the lower portion (/segment) was comparable between the two groups (Fig. 4B). Likewise, the functioning volume of the upper portion (/segment)—the functioning volume of the lower portion (/segment) was comparable between the two groups in patients undergoing lower lobectomy (Fig. 4C).

## DISCUSSION

The use of chest CT data in preoperative risk assessment for patients with resectable lung cancer is reasonable because whole-lung CT is routinely performed in preoperative oncological assessments. In addition, CT data can be obtained from patients who are unable to undergo spirometry. Several reports have shown that %LAA is predictive of postoperative complications [16–22]. However, none of these reports compared the predictive values of ppo%FEV1, ppo%DLCO and %LAA. In the current study, we clarified that the combined use of %LAA and %DLCO was useful for preoperative risk assessment in preoperative patients undergoing major lung resection for lung cancer. Thus, %LAA can be used as a substitute for ppo%FEV1, particularly in patients in whom the measurement of FEV1 is difficult.

We believe that the use of %LAA is beneficial because it does not involve any additional cost and requires minimal labor and time: %LAA can almost be automatically obtained using an imaging analysis software program. It is known that both FEV1/FVC and %LAA can be used to grade chronic obstructive lung disease [25]. However, FEV1/FVC represents airflow obstruction, and %LAA represents alveolar wall destruction; thus, there is only a slight correlation between the two parameters. In the current study, a correlation analysis showed that %LAA was only weakly correlated with FEV1/FVC and had no apparent correlation with other factors (Supplementary Fig. S1A–F). Therefore, we consider that it is appropriate to concurrently analyze ppo%FEV1 and %LAA in the multivariate analysis.

According to the relevant literature, a higher %LAA is associated with postoperative hypoxia, probably due to micro-atelectasis [12], prolonged postoperative air leak [14], development of lung cancer [26] and poor overall survival after lung cancer surgery [27, 28]. In particular, %LAA is a strong predictor of prolonged postoperative air leak, and a previous report suggested that the AUC for %LAA in predicting prolonged air leak was as high as 0.85 [29]. Thus, it remains unclear whether %LAA is a useful predictor of complications other than prolonged air leak. Therefore, we performed an ROC analysis to show the value of %LAA and %DLCO in predicting prolonged air leak (Supplementary Fig. S2A). The scatter plot of %LAA vs ppo%DLCO shows the distribution of patients who experienced prolonged air leaks (Supplementary Fig. S2B). These figures show that %LAA is not necessarily a strong predictor of prolonged air leak, suggesting that %LAA has the potential to predict both prolonged air leak and other complications. This discrepancy between the current study and the previous report may be due to technical improvements in controlling intraoperative air leak using the suture-and-reinforcement method [30]. In contrast, the reason why %LAA predicts complications other than prolonged air leak remains unclear. We believe that the reason may be multifactorial. For instance, patients with higher %LAA are likely to have a thin figure, low body mass index and sarcopenia, which are reported to be risk factors for postoperative CPCs after major lung resection [31].

In contrast to the negative effect of %LAA, some patients with moderate to severe emphysema may benefit from major lung resection because of the volume reduction effect. Our previous study suggested that severity of emphysema and functional residual capacity were useful to identify patients who may likely benefit from major lung resection [32]. Thus, comprehensive assessment of pulmonary function may be effective in patients with moderate to severe emphysema for therapeutic decision-making.

CT-based anatomical assessment is advantageous for evaluating the regional pulmonary function. In the current study, we quantitatively evaluated the distribution of emphysematous areas in the upper and lower lobes, in addition to the size equality between the upper and lower lobes on CT. As a result, lung heterogeneity did not affect the occurrence of postoperative complications. We believe that the lack of statistical significance may be due to the relatively small number of patients with heterogeneous lungs. Thus, careful assessment of regional lung function with CT, in combination with pulmonary ventilation/perfusion scan, is still needed in patients with marginal pulmonary function.

The present study was associated with several limitations. First, a selection bias could have influenced this study because surgeons may have refrained from major lung resection in patients with a poor pulmonary functional reserve but not in patients with severe emphysema. Supplementary Figure S3 shows a scatter plot of ppo%FEV1 vs ppo%DLCO. Note that postoperative complications were rarely seen in high-risk areas (ppo%FEV1 < 50% and ppo%DLCO < 50%). Second, we did not perform quality control of CT; the LAA can be accurately identified on CT and accurately corresponds to pathologically diagnosed emphysematous areas if CT is performed during a deep inspiratory breath hold. To improve the diagnostic accuracy of %LAA, spirometry-gated breath-holding CT is desirable. Third, the current study was a single institutional retrospective study. The cut off value for DLCO and %LAA should be analyzed and confirmed by a prospective multi-institutional study to determine if they can be used in clinical practice.

In conclusion, measuring the extent of emphysema on CT, in addition to ppo%DLCO, may contribute to accurate risk assessment in patients undergoing surgery for primary lung cancer. %LAA can be used as a substitute for ppo%FEV1, particularly in cases in which FEV1 is difficult to measure.

## Supplementary Material

ivaf027_Supplementary_Data

## Data Availability

The data underlying this article will be shared on reasonable request to the corresponding author.

## ABBREVIATIONS

AUC: Area under the ROC curve
CPC: Cardiopulmonary complication
CT: Computed tomography
DLCO: Diffusing capacity of the lungs for carbon monoxide
FEV1: Forced expiratory volume in 1 s
%LAA: Low-attenuation area
ppo%DLCO: Predicted postoperative DLCO
ppo%FEV1: Predicted postoperative FEV1
ROC: Receiver operating characteristic
SD: Standard deviation
