# Supplementary material for: Significance of measuring the severity of emphysema, in combination with spirometry, on the risk evaluation of patients undergoing major lung resection for cancer
Source: Interdiscip Cardiovasc Thorac Surg. 2025 Feb 11;40(3):ivaf027. doi: 10.1093/icvts/ivaf027 (PMC11890287; doi:10.1093/icvts/ivaf027)
Supplement: ivaf027_Supplementary_Data [file ivaf027_supplementary_data.zip › Supple figure legend.docx]

Supple figure legend

Supplementary figure 1

Correlation between %LAA and various factors.

Supplementary figure 2

Scatter plot of %LAA and ppo%DLCO (A). Patients with prolonged air leak are shown in red solid circles. Note that patients with prolonged air leak are not remarkably concentrated in higher %LAA area. ROC curves showing potential of %LAA and ppo%DLCO for predicting prolonged air leak (B). The area under the curves for %LAA and ppo%DLCO are comparable to those for %LAA and ppo%DLCO for predicting any postoperative complications, as shown in figure 1.

Supplementary figure 3

Scatter plot of ppo%FEV1 and ppo%DLCO, showing the distribution of patients with postoperative complication (red circle). Note that postoperative complications are rarely seen in conventional high-risk area (ppo%FEV1 < 50% and ppo%DLCO < 50%).
