# Supplementary figures and images for: Significance of measuring the severity of emphysema, in combination with spirometry, on the risk evaluation of patients undergoing major lung resection for cancer
Source: Interdiscip Cardiovasc Thorac Surg. 2025 Feb 11;40(3):ivaf027. doi: 10.1093/icvts/ivaf027 (PMC11890287; doi:10.1093/icvts/ivaf027)

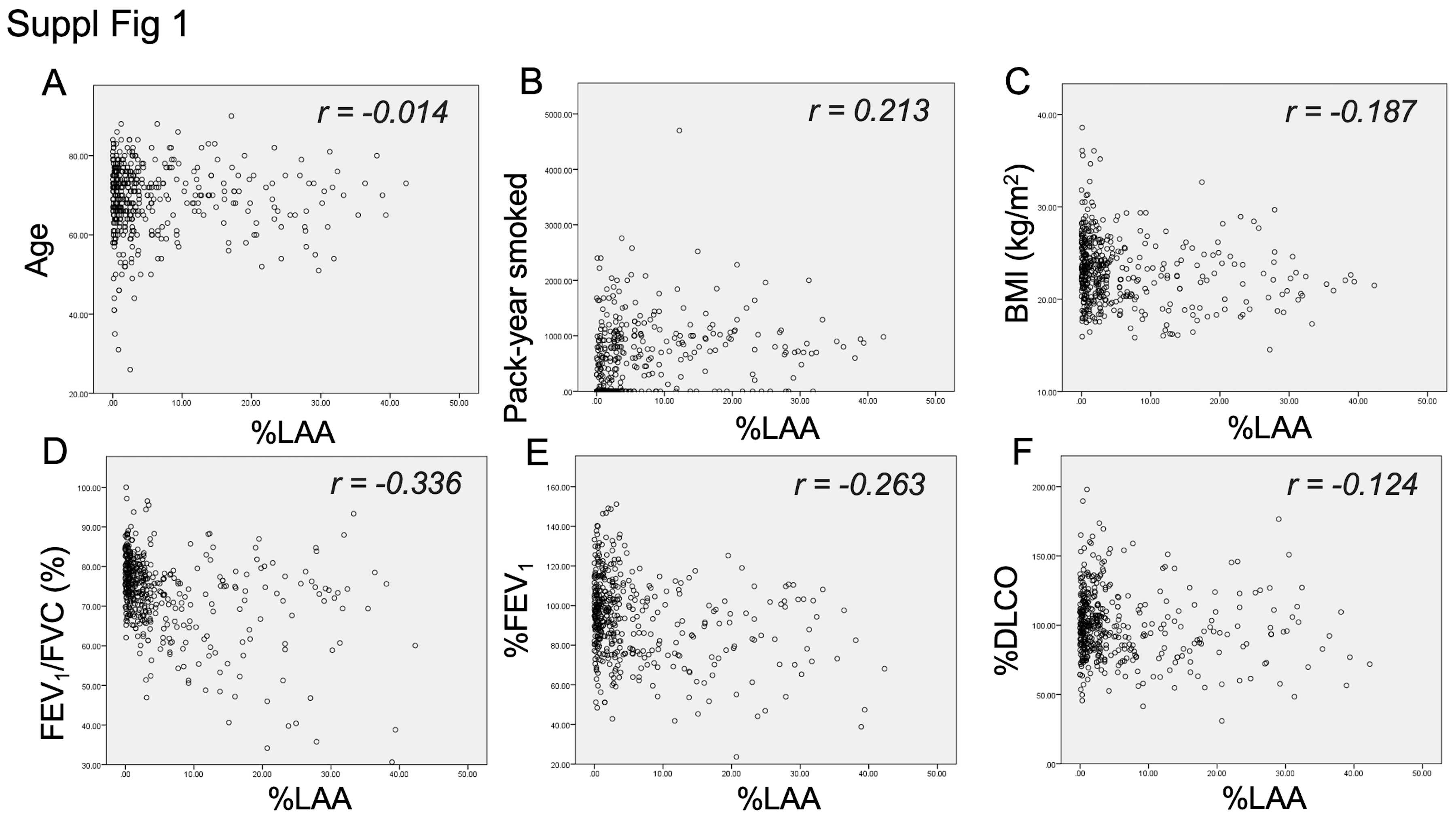

Supplement: ivaf027_Supplementary_Data [file ivaf027_supplementary_data.zip › SUPPL FIG1.tiff]

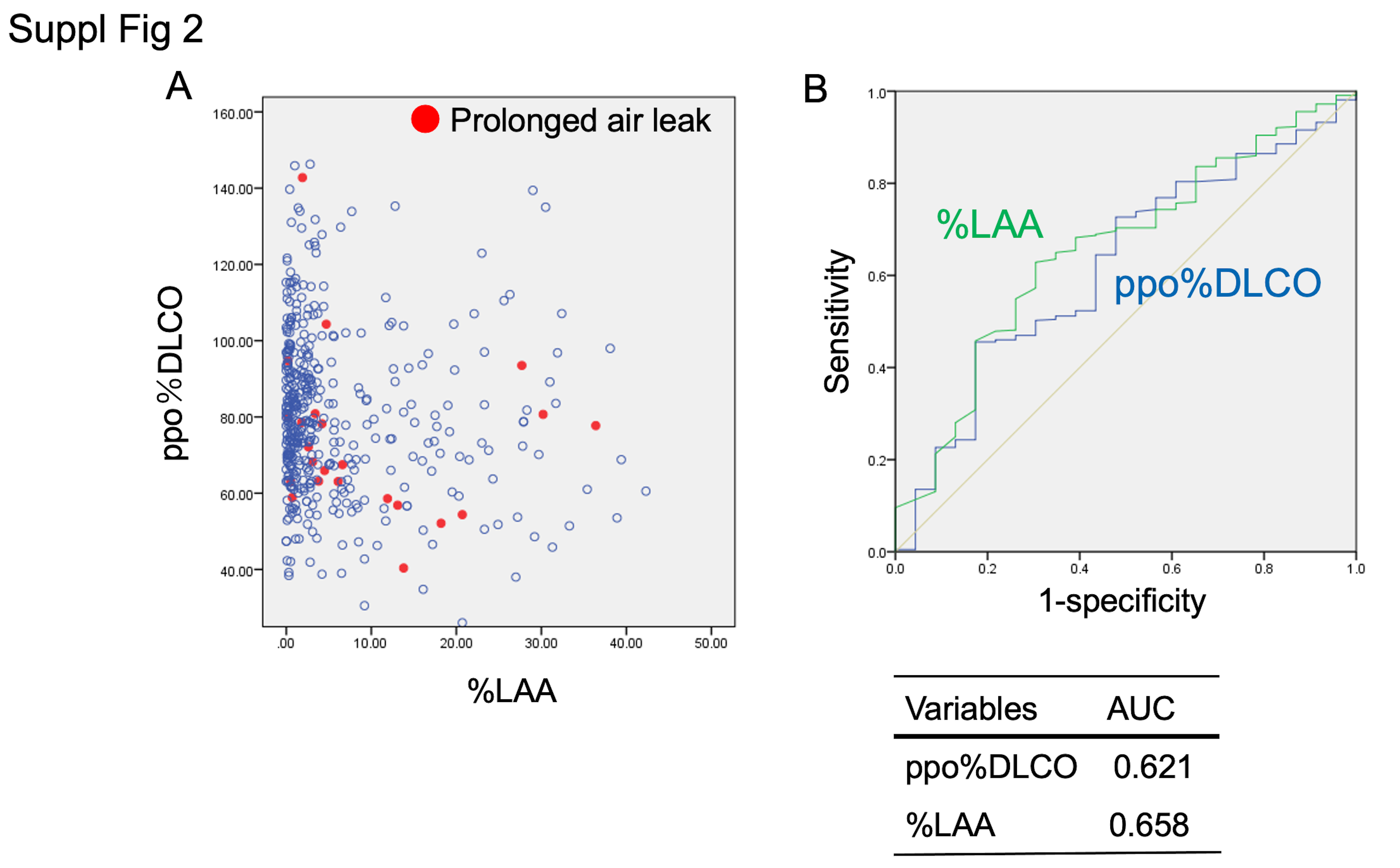

Supplement: ivaf027_Supplementary_Data [file ivaf027_supplementary_data.zip › SUPPL FIG2.tiff]

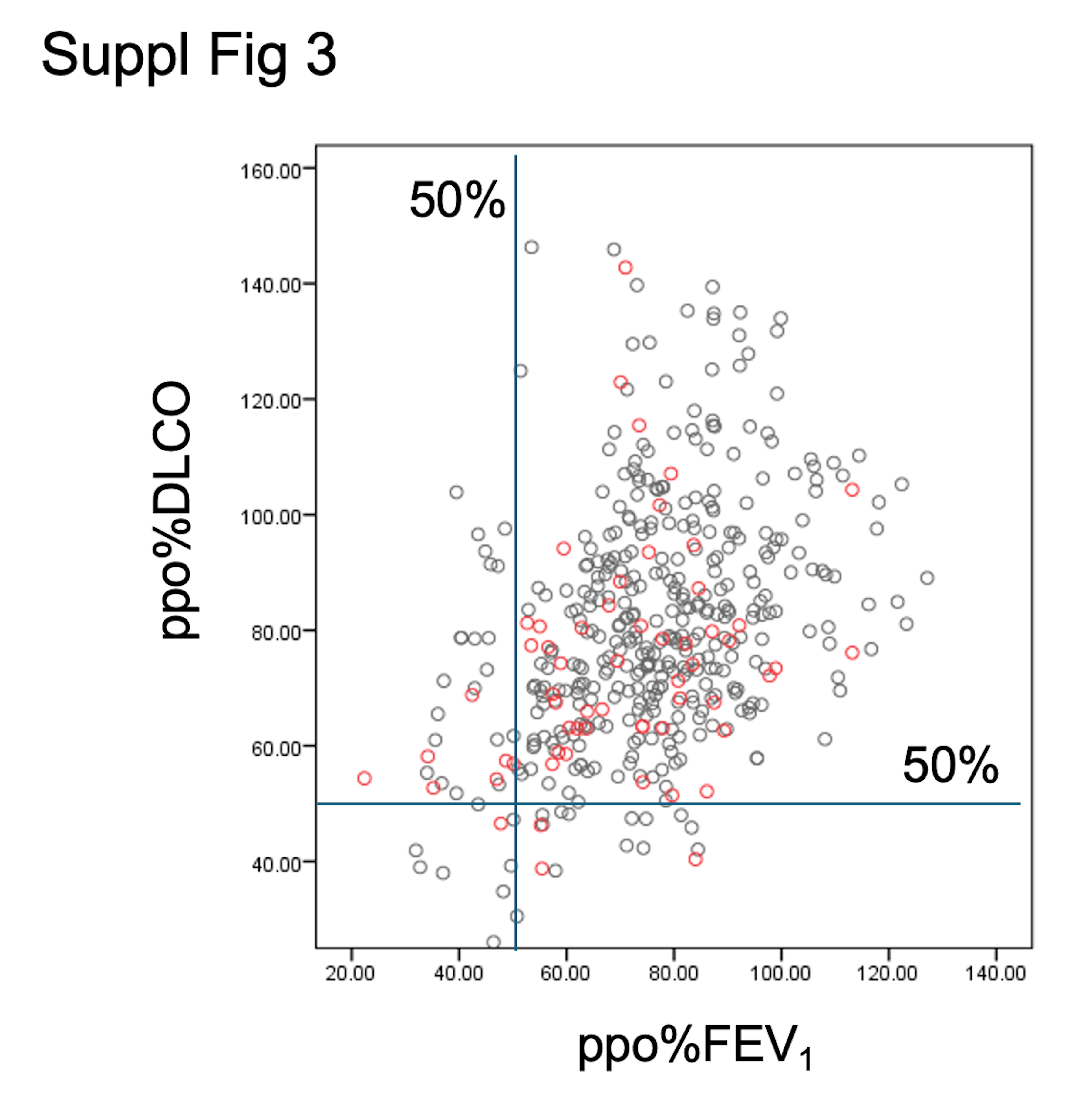

Supplement: ivaf027_Supplementary_Data [file ivaf027_supplementary_data.zip › SUPPL FIG3.tiff]
